# A History of Ileo Colitis

**Published:** 1854-12

**Authors:** S. W. Thompson


THE NORTH-WESTERN

MEDICAL AND SURGICAL JOURNAL.

NEW SERIES.

VOL. III. DECEMBER, 1854. NO. 12.

### ORIGINAL COMMUNICATIONS.

ART. I.—*A History of Ileo Colitis*, as it prevailed in and around Fairfield, Wayne county, Illinois, during the summer of 1854. By S. W. Thompson, M.D.

Gentlemen—Deeming, as I do, that it is a duty imperative upon every physician to record the progress of every epidemic visitation of disease, of whatever kind it may be, or whatever character it may assume at the time of such visitation, I have thought it might not be uninteresting to you were I to give a brief sketch of the disease at the head of this article, as it prevailed during the past summer in the county where I reside. I may have been mistaken in my diagnosis of the actual morbid anatomy present; as also I may have been in the mode of practice which I thought it advisable to pursue; I may be wrong in some of the reflections and ideas which I shall here offer. But of these things you must be the judges, and should such be the case, some of you, as well as myself may learn better by seeing my errors. But to proceed.

The disease first showed itself in a serious epidemic form about the middle or last of June, 1854, and continued on with little or no abatement of its virulence until the middle or latter part of

August following. What I wish more particularly to call your attention to in this paper, is the peculiarly low and malignant character assumed by the disease in question; its little amenability to ordinary treatment, and the *early appearance* of a number of grave symptoms, usually occurring after a somewhat lengthened sickness. That there has been with us this summer a very general tendency to low or adynamic stages of disease is too well known to require further notice. What the true reasons of this disposition are, is still a mystery in part. We may if we please attempt to ascribe it to the excessively hot and dry summer appearing immediately after a cool and very wet spring. But we are still no nearer an explanation when we find that counties adjoining Wayne and subject to the same meteorological influences, and not differing materially in the soil, remained almost entirely exempt from any serious epidemic disease for at least two months later than we did. And still more are we convinced of the insufficiency of any such explanation, when it is known that although the tendency to a low grade of disease, especially of the character of the one now under consideration, was generally manifested throughout Wayne county, yet in certain small and well-defined localities alone did there appear that peculiarly epidemic and malignant tendency of which I wish more particularly to speak.

Having prefaced by these brief remarks, I shall endeavor to describe the disease in detail as I witnessed it myself and heard it described by trustworthy and competent physicians.

The first serious case I recollect seeing was that of a man of somewhat weakly habit. He had complained of suffering from the excessive heat, and had some slight and transient diarrhœa occasionally for a fortnight, when about the first day of July he was seized with all the symptoms of dysentery. Fever, tenesmus, tormina, pulse quick and rather hard and small, not full. Stools consisted of mucus mixed with blood and a little fecal matter. Some tenderness over the region of the colon; tongue slightly coated. I did not see him at this time, but received the above account from another physician. On the morning of the fifth of July I was called in consultation. The case was then desperate, indeed hopeless. There was considerable tormina, tenesmus, and fever; tongue was coated brown, and cracked; sordes upon the teeth; pulse small, frequent, weak and fluttering; considerable emaciation; lay in a state of stupor, partly I think the effect of opium previously given; stools were frequent and composed of greenish bloody water, sometimes almost clear blood, and at other times containing some amount of mucus. Upon pressure in the right iliac region, I discovered some tenderness, and this upon slight pressure could be traced over the region of the small bowels. The pain upon pressure in the course of the colon was not very acute. Partial perspiration would now and then appear; patechia were already plainly manifested over the chest, neck and arms. Sub-sultus tendinum was also present to a marked extent. As whatever was to be done had to be done promptly, the resisting powers of nature being rapidly upon the decline, there was administered Quinine, hydrarg. cum creta, and Dover's powder, along with turpentine. Also glysters of gum arabic emulsion and turpentine. These means entirely failed to check the discharges from the bowels, and although he appeared for a short time to revive under the stimulation of the turpentine, there shortly after appeared vomiting of the same character of matter as thst passed by stool. Astringent injections, quinine, wine and turpentine, were again administered without avail, and he died on the afternoon of the seventh. From the first time I saw him, the stools were very offensive, but towards the last day they assumed the appearance of decomposed blood, and smelt so intolerably offensive as to sicken the attendants. There was present some disposition to retention of urine, but it was not serious, and I thought might be caused by a large blister over the abdomen, which had been applied and kept dressed with mercurial ointment before I saw him. This patient, towards the last few hours of life, became dull, indeed almost comatose.

The next case I saw was that of T. R., æt. 18. He was attacked on the 6th of July with tormina, tenesmus, and passage of mucus sanguinolent stools. Tongue somewhat coated with a whitish fur; some headache, but little fever; great tenderness in the track of the colon. His bowels had previously been regular. I immediately gave—

| | | |
|---|---|---|
| Calomel, | . . | gr. xxiv. |
| Dover's Pulv., | . . | gr. vi. |
| Opii, | . . | gr. iv. |
| Plumbi acet. | . . | gr. xv. |

in cht. iii., one every four hours.

The patient was ordered to keep quiet, and use demulcent drinks. Under this treatment he improved rapidly for a couple of days; but at the end of that time he exerted himself too much, and was taken with a relapse. The mode of treatment pursued this time was similar to the above, but substituting more opium and ipecac., and less of the sub. mur. He again began to improve, but had some flashes of fever. I now applied a large blister over the abdomen, and substituted the hydrg. cum creta, opium and plumbi acetatis, for the medicine previously given. The blister drew well, and was followed by a decrease of all the bad symptoms. Tongue looked clean; bowels were moved only to a moderate extent, and the stools began to assume their natural color and snbsistence. Urine high-colored and copious, depositing considerable sediment upon standing. There had been some retention of urine; but this symptom was now relieved. Pulse felt soft and slow; no headache, no fever. The tormina and tenesmus had entirely disappeared. He continued to improve until the night of the 11th, when he was taken suddenly worse. There was some return of tormina and tenesmus, with pain upon pressure in the right iliac region. Fever, with a very harsh and dry skin; pulse weak. quick and soft. I had ordered a dose of castor oil to be taken the evening before, but it had been neglected. I now again ordered the same, with the addition of ʒss of turpentine. He had frequent bloody, greenish, watery stools, the blood being intimately blended with the rest of the matters passed. Tongue coated brown, dry and cracked. There was present some muttering delirium, and sub sultum tendinum. I ordered, after the oil and turpentine were taken,

| | | |
|---|---|---|
| Hydrg. cum creta, | . | gr. xv. |
| Morphia sulph., | . . | gr. ij. |
| Quinine, | . . . | gr. viii. |

in cht. ij., one every six hours.

On the next day he was about the same. The oil brought away one dark feculent discharge, and then the passage of greenish bloody, and very offensive watery stools continued. There was some slight disposition for the tongue to clean off. Still some fever, but extremities are sometimes cold. Moans and talks in his sleep. Great restlessness. The tenderness and gurgling in the right Iliac Fossa continued and seemed to be increasing. Ordered the surface to be sponged with cold water and seidlitz powders to be taken pro re nata. Also,

Calomel, gr. vi,
Opium, gr. ij,
Quinine, gr x.
in cht iij, 4 to 6 hours.

Repeat Castor oil and Turpentine. On the 13th I found him some better. The oil and turpentine brought away some two or three dark feculent discharges, not nearly so fetid or bad colored as previously. I now ordered

Dover's powder, gr. xv,
Opium, gr. iij,
Ext. Gentian, f s,
Quinine, gr. xx.
In pills, viii, 2 every 4 hours.

Spts. Etheris Nitrici ℥j coch. parv. 2 hours. Sponging the surface with warm vinegar and water.

After this date he continued to improve, and he took no medicine except Quinine and Dover's powder.

His family afterwards removed him to a distance of twenty miles, but he did not seem to suffer materially from fatigue, and eventually slowly recovered.

I saw several other cases about this time, and all were taken in a way similar to those just described, and in all at the end of two or three days if the disease was not previously checked, there came on purging of bloody or greenish bloody water, with pain and gurgling in the right Iliac Fossa, and in many there was present general abdominal tenderness with Tympanites. The Tormina and Tenesmus, and passage of more or less Mucus continued after symptoms of inflammation of the small intestine came on. In some there would be a greater predominance of symptoms referable to the colon, than in others. But it would be tedious for me to give you a history of each individual case, and I shall therefore relate but one more in detail.

On the 28th of July I was called in consultation to see a lady affected by this disease. The symptoms previous to my seeing the patient were as follows. She was taken suddenly three days be fore, having previously been in good health, with violent and continued pain in the course of the colon, with the passage of blood and mucus. Some considerable fever. Pulse quick and full, but not very hard. Tongue coated to a moderate extent with a yellowish white fur. Extremities at times inclined to be cold. Tender ness upon pressure, in the track of the colon. Considerable Tenesmus. At the time I saw her the symptoms were as follows. Paroxysms of pain through the bowels, more especially upon the passage of stools, which occurred every half hour or hour. High fever, but extremities occasionally cold. Some headache. Tenderness and gurgling in right Iliac fossa. Stools passed involuntarily, and were of a pinkish green offensive water, mixed with small clots of partially decomposed blood, and a considerable amount of mucus. Pulse very quick, weak and thready, yet not very soft. Marked Sub sultus Tendinum. Tongue dry, coated, brown and fissured. The patient lay as if wishing to remain undisturbed, except during a paroxysm of pain, when she cried out for some one to help her. Petechia already apparent on the breast and neck. There was also over the same part, a slight vascular eruption, the vesicles of which, soon after they had acquired the size of millet seeds, became filled with an opaque serous fluid, having somewhat the appearance of thin pus. They shortly became depressed in the centre, and they assumed every appearance of Small Pox pustules. Whenever they were ruptured, they poured out the contents and left nothing but minute red points.

The plan of treatment pursued previously to my seeing her and continued in after that time, was the administration of alterative doses of Hydrg. Sub. Mur, combined with opium, Ipecae and Quinine. Sponging the surface with cold water to relieve fever. Gum Arabic Emulsion, Turpentine and Laudanum given by the mouth and also by glysters. Wine was allowed pro re nata. She continued to grow worse. The vesicular eruption previously spoken of continued to increase, as quickly as one crop disappeared another showing itself. The stools continued about the same, of a most offensive character and still passed involuntarily. Pulse weaker and more rapid. Retention of urine also occurred, but was relieved by an emolient poultice to the Hypogastrium. Fever about the same, some wandering and delirium. Tongue brown and cracked. Sordes on the teeth. Tenderness along colon still continued, as did the tenderncss and gurgling in right Iliac fossa. Abdomen became Tympanitic, and extremities gradually cold. I applied a blister to the abdomen which drew tolerably well, but afforded no relief. The hands and feet were bathed in stimulating linaments with a temporary restoration of warmth. Quinine, Hydrg. cum Creta and Dover's Powder were continued along with wine by the mouth and injections. Aqua Camphora or Spts: Mindareris given pro re nata. Light and nourishing food ordered, as there was evidently a rapid failing of the resisting powers of nature. Vomiting of a greenish, watery and very offensive matter took place shortly after. Injections of wine and beef tea ordered to be continued; but it was all to no purpose. She continued to *exist* for several days in a state at times approaching to coma. The subsultus Tendinum &c. continued to the thirteenth day when she died.

The above are true descriptions of almost all the bad cases of the disease which presented themselves. They all began as Dysentery, but rapidly degenerated into a grade of disease, the symptoms of which in most things exactly accord with our knowledge of severe cases of Typhoid fever. But there is this marked difference between the two. In the disease we are now discussing, there was always at the commencement symptoms of severe Colitis, and these continued with more or less severity to the end. This is not the case in Typhoid fever. There is another important difference which it will be well to mention, viz. The early appearance of Petechia, Sudamina and Sub Sultus Tendinum, which usually appeared *from the third to the fifth day, and in some from the first of attack*, whereas in Typhoid fever we look for parallel symptoms not before the eighth day. The first and last cases related above were the only fatal ones coming under my own observation, but the fatality generally in the localities where it prevailed epidemically was truly melancholy.

As I remarked in a previous part of this paper, there are many points of resemblance between this disease and Typhoid fever. The purging of liquid stools, in many cases nearly pure blood; the disposition to low muttering delirium and general prostration, along with the occurrence of sub sultus and Patechia, would all point out the similarity. But there are still other symptoms which lead us to note this connexion still more closely. These are, first, the evidences apparent of a limited contagious nature possessed by the epidemic, and secondly, its being confined with a few exceptional cases to certain well defined localities. That the disease in question was to a limited extent contagious presents itself strongly to my mind. That it occurred independently of such cause is beyond doubt; yet the occurrence of the disease could in many instances be traced so directly to contagion, that it would be difficult to entirely throw away such evidence, even were it in direct opposition to our usually received opinions. I will mention one instance in point. The third case related in this paper, had been, so far as could be ascertained, subject to no contagious influences for some time. But three of those who were in constant attendance were attacked; two of these resided when at home in districts not subject to the disease. One of the cases proved fatal. The child of the patient in question was seized with it and died. Her mother-in-law and sister, who, besides those mentioned above as having contracted the disease were in close attendance, were also attacked but recovered. I myself contracted the same complaint, but arrested it promptly by means of large doses of opium. This, it is true is but a solitary instance, yet others occurred of almost as marked a character as the above, and there was such an evident connection between effect and cause, that I think we are hardly justified in repudiating the idea of the disease in question being to a limited extent contagious. Nor is this idea at all novel. Our best authorities upon Typhoid fever, argue in support of the disease being more or less contagious in its nature. Many of the older writers believe dysentery to be contagious when occurring epidemically, and some of our best modern authors admit that it may be, and probably is so under certain peculiar circumstances; and, what I would ask, is so likely to give a contagious principle to dysentery as its complication with a Typhoid character of disease? Dr. Bell, although not subscribing to the doctrine of its contagious nature, says: "The most plausible argument in favor of the contagiousness of dysentery are made when the disease is associated with fevers of a Typhoid character and sometimes with Typhus itself." In the same paragraph he adds: "But in such cases dysentary, like bronchitis, is a superadded disease, a complication of the original malady, and in this view does not come within scope of investigation into the etiology of either sporadic or epidemic dysentery.

I cannot however, give an unreserved and unqualified assent to this doctrine, for it seems to me improper to make the colitis the superadded disease, when really it was the primary one. A typhoid character of fever may come on subsequently to the appearance and predominance of dysenteric symptoms, as did actually occur in the epidemic I am now recording; but certainly it would be far more proper to call the fever superadded, instead of attributing the cause to the effect, as would be done by reversing the matter as Dr. Bell intimates.

Another analagous feature to typhoid fever, possessed by the epidemic, and at the same time it is a slight evidence of its possessing a contagious character, is its confinement to certain settlements, or well defined localities or neighborhoods. This was so strictly true in Wayne Co., that except a very few scattering cases of the disease, it did not spread beyond the limits of such localities or neighborhoods as it first appeared in. For instance; in the town of Fairfield, there were few families which entirely escaped its visitation, yet here the severe cases were confined to one part of the village; the disease did not extend around the town, except in one or two instances where those so attacked had been in close attendance on others suffering from the complaint. Another neighborhood about four miles north-east of Fairfield, suffered even more severely than did the latter place; almost every case attacked here within a space of two miles square proving fatal; nor was this fatality confined to the practice of one physician merely, for all who had much to do with the disease in this neighborhood were nearly equally unfortunate.

From closely and attentively watching the symptoms and progress of this epidemic, I am convinced that the inflammatory process, in all cases, first commenced in the *colon*, but rapidly extended itself, until it reached the *small intestines*; in some cases involving the mucous coat throughout nearly their whole extent.—In the practice of Drs. Bell and Stokes we are told that dysentery is sometimes complicated with extensive disease in the small bowels; and in the opinion of these gentlemen, what is actually denominated typhoid dysentery, *is where the colitis is either complicated typhus or (typhoid) fever or extensive lesions of the small intestines.* It is most usual to see epidemics of "typhoid dysentery" in warm seasons and climates, and I would enquire, whether the extreme heat of the past summer might not be reasonably expected, under other favorable circumstances, to give rise to disease which would simulate those incident to tropical climates?

Upon the general treatment of the disease I shall be as brief as possible, confining myself to the practical details and abstaining from theoretical discourses and abstract ideas. It was only during the first few hours of atttack that much immediate benefit could be expected from the use of medicine. If therefore I saw at this period, I gave pretty free doses of opium combined with blue mass. The following was what I usually prepared for an adult.

℞ Pil. Hydrg. - - - ℈ij

Pulv. Opii - - - gr. xii

In pills x. 2 at once and 1 every 4 hours until the discharge ceases.

In some cases where the patient had been in good health, young and robust, I preferred to use the mild chlor. of mercury in place of the blue mass. I sometimes gave ipecac or pulv. comp., but the irritability of the stomach was generally so great as to preclude the use ofeither. I will here mention the peculiar susceptibility that existed to the scialagogue action of mercury. In some cases after giving a dose or two I have been compelled to desist from the use of this valuable alterant, and in others, quite a troublesome ptyalism supervened upon taking three or four doses of the remedy.

After giving the medicines mentioned above, should the bowels remain costive, I usually give a dose of castor oil and laudanum, and this in a large majority of instances was all the medication required, if the patient was seen in the commencement; but should the disease be allowed to go unchecked, it became necessary to resort to other remedies. When therefore the tongue became dry, brown and cracked; the pulse weak and irregular; when there was present fever, low muttering delirium and sub sultus, and at the same time the discharges from the bowels are bloody water, mixed with mucus, I trusted to the administration of quinine, opium and hydrg. cum creta. Spts. etheris nitrici given freely. Small quantities of quite cold water for drink, and sponging the whole surface with warm or cold water to suit the patient's fancy. Glysters I have found this season to be of little or no benefit, except in cases where there was great nervous excitability and restlessness; when a simple warm anema of gum assafœtida in water or thin starch proved of great service. Should the abdomen become tympanitic, a cloth wrung out of hot water and sprinkled with turpentine I have found to be a most admirable topical application to the bowels. Great benefit may be expected from blisters, but care is required to watch the proper moment for their application, and I am satisfied that in one or two instances I have seen harm result from their too early use. When the most severe symptoms, such as tormina, tenesmus, and discharge of bloody stools, had somewhat but not entirely ceased; when the fever was disposed to become remittent in its character, or of a lower grade than it previously had been; or when the extremities were disposed to become cold, I have found to be the most advantageous time for their application. They should be kept on until their epispastic effect is sure of being obtained, but not longer, as in the latter case the irritation excited by them may be so great as to neutralize the good effect of their derivative action.

About the same time that blisters are admissible, turpentine also effects its most valuable results. This remedy I have given in doses of from ten drops to half a drachm every two hours, and with the most marked benefit. I usually combined it with laudanum; and the administration has been followed, in many instances, by a decreased and improved condition of the discharges from the bowels; a relaxation and softening of the skin, and clearing off of the tongue. I cannot give minute directions concerning all the little "et ceteras" required in the treatment of this or any other disease; as such things must, of course, be filled up by the good sense and judgment of the physician. I will say a word, however, upon the use of quinine in this disease. *I have used it in every case;* sometimes from the very beginning; at others not until the latter stages of the complaint. In regard to the time of its administration, I have been governed entirely by the character of the attack. If it was one in which I might reasonably expect the rapid supervention of typhoid symptoms, I commenced giving this remedy at once; otherwise I waited for the appearance of symptoms indicating its use. The administration of wine or brandy was governed by nearly the same circumstances—of course desisting from their use as long as possible, should there be high fever, with great abdominal tenderness. I did not use cups, as I did not meet with cases wherein I thought them applicable; but I knew of several cases in which they were applied, but without any benefit resulting from their use.

I have thus given a succinct, yet, I am aware, an imperfect history of the disease which so sorely afflicted the community where I reside, and the progress of which was so well calculated to make the physician doubt the boasted powers of medicine, and cause him to dive still deeper into the mysteries of disease, and try to fathom its utmost depths. Medicine seemed, in a very large proportion of cases, to have entirely lost its controling power over disease; and Death stalked, with all his nakedness, hand in hand with the dreaded pestilence. Should what I have here recorded be the means of enabling any one hereafter to treat the disease with better success than has been my melancholy duty to record, I shall be fully satisfied.

I doubt not that many opinions I have advanced in this paper will not meet with your approval; but of this I am not the master; and should further investigation convince me of my errors, if such I have been guilty of, I shall be most happy to acknowledge them.

